# Synthesis of NdAlO_3_ Nanoparticles and Evaluation of the Catalytic Capacity for Biodiesel Synthesis

**DOI:** 10.3390/nano9111545

**Published:** 2019-10-30

**Authors:** Mayra Dionicio-Navarrete, C. Dinorah Arrieta-Gonzalez, Alfredo Quinto-Hernandez, Maura Casales-Diaz, Jacqueline Zuñiga-Diaz, Jesus Porcayo-Calderon, Lorenzo Martinez-Gomez

**Affiliations:** 1 Instituto de Ciencias Físicas, Universidad Nacional Autónoma de México, Avenida Universidad s/n, Cuernavaca 62210, MOR, Mexico; mayra.dbnavarrete@gmail.com (M.D.-N.); mcasales@icf.unam.mx (M.C.-D.); lmg.icf.unam@gmail.com (L.M.-G.); 2 Tecnológico Nacional de México—Instituto Tecnológico de Zacatepec, Calzada Instituto Tecnológico 27, Zacatepec 62780, MOR, Mexico; cdaglez@gmail.com (C.D.A.-G.); aquintoh@yahoo.com (A.Q.-H.); jacque_zd@hotmail.com (J.Z.-D.); 3 CIICAp, Universidad Autónoma del Estado de Morelos, Avenida Universidad 1001, Cuernavaca 62209, MOR, Mexico; 4 Corrosion y Protección (CyP), Buffon 46, Mexico City 11590, Mexico

**Keywords:** NdAlO_3_, nanoparticles, canola oil, heterogeneous catalysis, transesterification

## Abstract

Biodiesel synthesis was carried out via heterogeneous catalysis of canola oil with nanoparticles of a mixed oxide based on rare earths. The catalyst synthesis (NdAlO_3_) was carried out based on the method proposed by Pechini for the synthesis of nanoparticles. Thermogravimetric analysis-differential thermal analysis (TGA-DTA) analysis was performed on the nanoparticle precursor gel in order to establish the optimum conditions for its calcination, with these being of 800 °C over 24 h. A pure NdAlO_3_ compound with an approximate size of 100 nm was obtained. The products of the transesterification reaction were analyzed using gas chromatography, FTIR, and NMR. The optimum reaction conditions were determined, namely, the temperature effect, reaction time, methanol:oil mass ratio, and recyclability of the catalyst. These studies showed the following optimal conditions: 200 °C, 5 h, methanol:oil mass ratio of 6:1, and a constant decrease in the catalytic activity of the catalyst was observed for up to six reuses, which later remained constant at around a 50% conversion rate. The maximum biodiesel yield obtained with the optimum conditions was around 75%. Analysis of the reaction products showed that the residual oil showed a chemical composition different from that of the source oil, and that both the biodiesel and glycerol obtained were of high purity.

## 1. Introduction

Pollution caused by the use and abuse of fossil fuels has created motivation for the search for sustainable energy alternatives. In this sense, undoubtedly, biodiesel is a real alternative that can be used to reduce mineral diesel consumption [1]. Biodiesel, although it can also be obtained from animal fat, is a derivative of vegetable oils, and therefore is a fuel friendly to the environment. Because biodiesel is non-toxic, biodegradable, free of sulfur, free of carcinogenic compounds, and contains oxygen in its molecule, it can be considered a clean fuel [2,3,4,5].

The use of biodiesel as an alternative fuel to fossil fuels has a direct impact on the reduction of CO_2_ emissions because its combustion emits the anthropogenic CO_2_ that plants used for their growth [6,7,8]. In addition, during the combustion of biodiesel, the energy obtained is actually solar energy that was used by the plants to carry out their photosynthesis activities: this is an additional feature to be considered as a clean fuel that contributes to reduce the greenhouse gas emissions [9,10].

Biodiesel synthesis can be carried out using a process of esterification and/or transesterification of vegetable oils, depending on their content of free fatty acids [11], in the presence of a catalyst, where the catalytic reaction can be homogeneous or heterogeneous.

Most of the research has been focused on the study of the transesterification reactions of vegetable oils based on homogeneous systems due to their high yield and lower reaction time [1,12]. The most commonly used homogeneous catalysts are based on methanolic solutions of NaOH and KOH, which, when mixed with the oil, form a single phase that favors the contact of the reaction mixture, minimizing the mass transfer resistance, and therefore increases the reaction rate. However, the main disadvantages of this system are: use of raw materials of high purity (no presence of moisture and low content of free fatty acids), catalyst is corrosive and non-recoverable, uses many stages of purification to obtain a high-quality biodiesel, and the purification of crude glycerol is not profitable [1,12,13,14,15,16,17,18].

In the case of the use of heterogeneous catalysts, the reaction mixture forms an immiscible system (oil–methanol–catalyst) that faces many mass transfer problems that cause low reaction speeds and lower biodiesel yields [19]. However, the main advantages are: recovery and reuse of the catalyst, higher purity of the reaction products, less pollutant emissions to the environment, and lower production costs [1,4,12]. Unlike homogeneous catalysts, heterogeneous catalysts can be considered as green catalysts due to their easy recovery and recyclability [4,17,20]; in addition, many solid catalysts based on mixed metal oxides have shown a tolerance to the presence of free fatty acids and moisture [15].

Heterogeneous catalysis can use three types of solid catalysts, namely those of an acidic character, those of a basic character, and bifunctional ones (acid-base character) [3,4], with the basic ones being the most used due to their higher reaction rate [17]. Many studies have shown that rare earth-based oxides can be used to carry out the esterification and transesterification of vegetable oils [1,4,9,12,15,16,17,18,19,20]. The excellent catalytic activity of the rare earth oxides is comparable to that of CaO, and this is determined by its crystalline structure and cation radius [17,21,22,23]. However, one of its main disadvantages is the leaching of the catalyst, which reduces its stability and catalytic activity [12,17,18,24], in addition to the greater reaction time required to obtain performances comparable to those achieved in homogeneous catalysis [20].

Due to the various advantages offered by heterogeneous catalysts, and in particular those of a basic nature, the search for new catalysts for the transesterification reaction is important. Each heterogeneous catalyst behaves differently, which requires further investigation in order to determine the optimal reaction conditions to achieve the highest possible yields in the shortest reaction time, as well as in terms of the lowest temperature, and lowest use of methanol and catalyst [20]. In this sense, perovskite-type mixed oxides, based on rare earths, have greater chemical stability than simple rare earth oxides or rare earth oxides supported on other oxides (such as α-Al_2_O_3_). Its greater chemical stability is an advantage because its leaching can be significantly reduced, and therefore, its possible loss of activity is reduced.

Therefore, the purpose of this study is to explore the potential use of a NdAlO_3_ perovskite as a catalyst to carry out the biodiesel synthesis. For this, this study considers the synthesis and characterization of the NdAlO_3_ nanoparticles, and the evaluation of its catalytic activity in the biodiesel synthesis from canola oil. Nanoparticles were synthesized using the sol-gel process, and its catalytic activity was determined based on the methanol:oil ratio, temperature, reaction time, and catalyst recyclability. Reaction products were analyzed using FTIR, NMR, and gas chromatography.

## 2. Materials and Methods

### 2.1. Catalyst Synthesis

**Figure 1:**
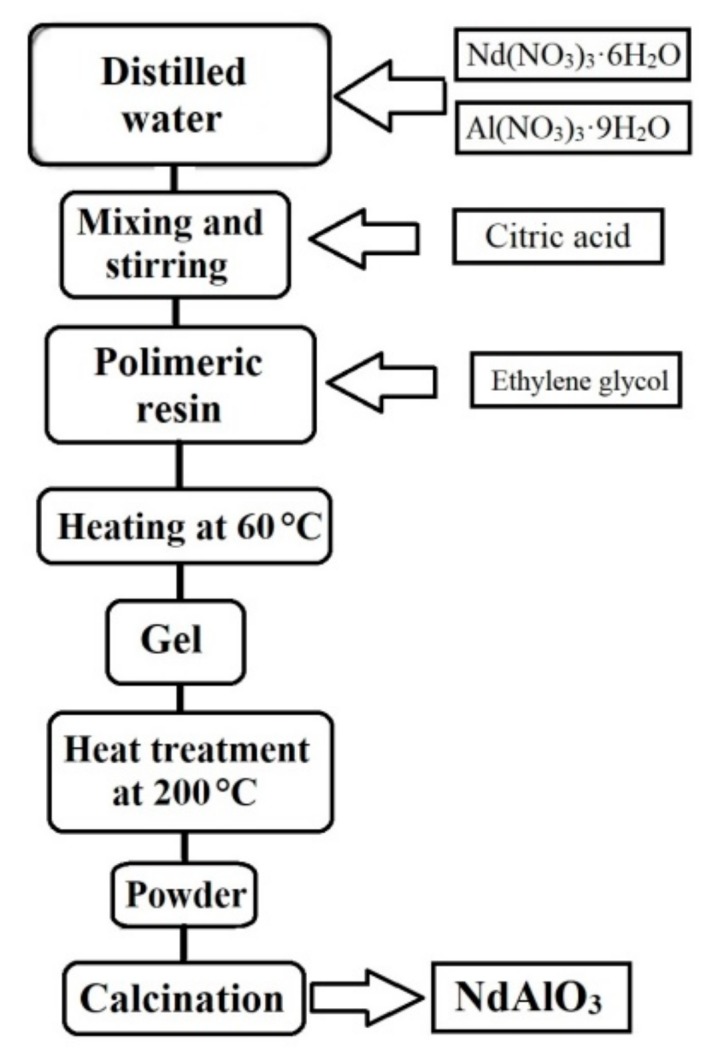
Scheme of the synthesis of NdAlO_3_ catalyst.

The catalyst synthesis was carried out based on the method proposed by Pechini for the synthesis of nanoparticles [25]. All other chemicals were purchased from Sigma-Aldrich (Saint Louis, MO, USA) and used without further purification. The synthesis process consisted of the formation of a polymeric resin due to the polyesterification between a metal complex (citric acid–metal ion) and a polyvalent alcohol, such as ethylene glycol. In this process, a homogeneous distribution of the metal ions into the structure of the formed resin was obtained, thus limiting its ionic mobility and its close contact. This spatial distribution favors the formation of a finely dispersed homogeneous phase after its calcination [26,27]. One of the main advantages of this process is its better control of the stoichiometry, lower reaction temperatures, less contamination, and better capability to prepare both ultrafine powders and thin films [27]. Figure 1 shows the process for the synthesis of the catalyst used in this work (NdAlO_3_).

The gel was characterized using thermogravimetry and differential thermal analysis (TG/DTA, TA Instruments, New Castle, DE, USA). Based on the TGA/DTA analyses, the gel was subjected to a pre-calcination treatment at 200 °C in an electric oven for 2 h, and finally calcined at 800 °C for 24 h in static air. The compound obtained after the calcination process was characterized using X-ray diffraction (XRD, Bruker D8 Discover AXS GmbH, Germany) and scanning electron microscopy (SEM, JEOL JSM-IT500, Jeol Ltd., Tokyo, Japan). The surface characteristics (specific surface area and pore size) of the catalyst were determined using the Brunauer–Emmett–Teller (BET) method using a chemisorption–physisorption analyzer (Quantachrome, Autosorb-1-C, Boynton Beach, Fl, USA). The determination of the basic strength and basicity of the catalyst was determined using the Hammett indicator procedure [4,28].

### 2.2. Catalytic Reaction of Transesterification

**Figure 2:**
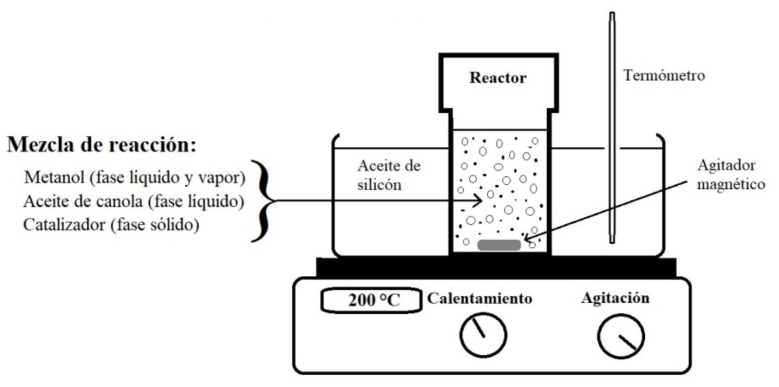
Schematic diagram of the experimental arrangement for the transesterification catalytic reaction of canola oil.

The catalytic activity of transesterification of NdAlO_3_ was checked using canola oil in a batch reaction system. The canola oil was of commercial edible quality and was used without any additional treatment. The transesterification reactions of the canola oil were carried out on a laboratory scale in a 100 mL stainless steel reactor. The reactants were poured into an airtight Teflon container that was introduced into the reactor. Canola oil (10 g), anhydrous methanol (in different methanol:oil mass ratios), and 1.0 g of catalyst (10% by weight with respect to the oil amount) were poured into the reactor. The amount of catalyst used was 10 times more than the amount of catalyst used in the homogeneous basic catalysis; however, the molecular weight of the heterogeneous catalyst (NdAlO_3_) was approximately 4 times that of the homogeneous catalyst (KOH). Silicone oil was used as a means of heating the reactor. The agitation of the reactive mixture was carried out with a magnetic bar at the maximum rotation rate of the heating plate (Figure 2).

After the established reaction time, the reactor was immediately cooled under a stream of water. Subsequently, the reaction mixture was subjected to a centrifugation operation (15,000 rpm, 20 min). The action of the centrifugal force allowed for the stratification of the different components of the reaction mixture, with the methanol–fatty acid methyl esters mixture being obtained in the upper part, which later contained the unreacted oil and the glycerol, and the catalyst was at the bottom. Methanol from the methanol–biodiesel mixture was removed via rotary evaporation to obtain the biodiesel. The conversion of oil to biodiesel was calculated according to the following equation.

Biodiesel yield (%) = [(Biodiesel mass obtained)/(Oil mass used)] × 100(1)

Under the experimental scheme described, different tests were carried out to test the effect of temperature and reaction time. For this, a fixed methanol:oil ratio of 2:1 (weight ratio) and a catalyst load of 10% by weight with respect to the oil mass were used. The test temperatures were 100, 150, and 200 °C. The test temperature was set in relation to the temperature of the heating oil (silicone oil). Previous tests in an instrumented reactor (not suitable to perform the synthesis) allowed for determining that the system reached the thermal equilibrium. The reaction times used were 0.5, 1, 2, 3, 4, and 5 h; For the determination of the optimum methanol:oil ratio, once the optimum reaction temperature was determined, the methanol:oil mass ratio was varied. The methanol:oil ratios used were: 2:1: 3:1, 4:1, 5:1, and 6:1. Regarding the transesterification kinetics, after determining the optimum conditions of temperature and methanol:oil ratio, the transesterification kinetics was determined as a function of the reaction time. The reaction times used were 0.5, 1, 2, 3, 4, and 5 h. Regarding recyclability of the catalyst, in order to determine the reusability of the catalyst, the procedure was as follows: after the transesterification reaction, the catalyst was separated from the reaction mixture using centrifugation, washed several times with acetone, dried at 80 °C for 4 h, and then calcined at 600 °C for 2 h in order to remove any organic matter residue. In this condition, the same catalyst charge was reused for the transesterification of the canola oil over 10 cycles. At the end of each cycle, the catalyst was subjected to the same separation and calcination process.

Both the oil and biodiesel were analyzed using FTIR spectroscopy, gas chromatography coupled to mass spectrometry (GC-MS), and nuclear magnetic resonance (NMR) in order to determine the characteristics and chemical composition of the reactants and products.

## 3. Results and Discussion

### 3.1. Catalyst Synthesis

**Figure 3:**
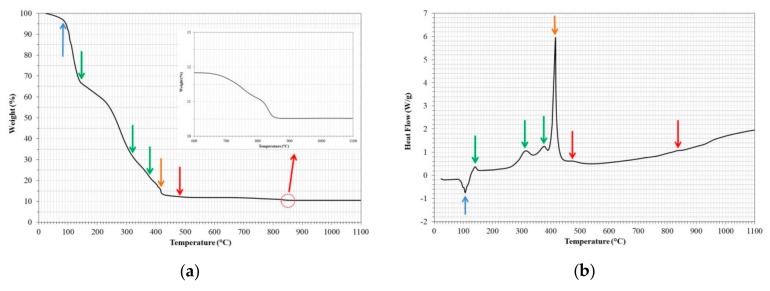
(**a**) Thermogravimetric (TGA) and (**b**) differential thermal (DTA) analysis of the precursor gel of the NdAlO_3_ nanoparticles (heating rate 5 °C/min in air).

Figure 3 shows the results of the thermogravimetric (TGA) and differential thermal analysis (DTA) performed on the NdAlO_3_ precursor gel, after a thermal treatment from room temperature to 1100 °C, at a heating rate of 5 °C/min in air. The polyesterification process of the metal chelate allowed for obtaining a homogeneous multimetal gel that was able to obtain the desired compound in aggregates of small crystallites of nanometric size during its thermal treatment [25,29]. During its calcination, the components of the polymeric gel behaved like oxidants and fuels releasing gases that favored the disaggregation of the products and the dissipation of the heat: this caused both an increase in the porosity and inhibited the sintering of the product [30].

According to the figure, it was an endothermic peak around 105 °C with a mass loss of approximately 5% (evaporation of adsorbed water). Subsequently, three small exothermic peaks were observed at 142 °C, 316 °C, and 377 °C that were associated with an additional mass loss of approximately 80% (breakdown of the polymer gel and release of water, carbon dioxide, and nitrogen oxides [31,32]). At 416 °C, a big exothermic peak associated with an additional mass loss of approximately 2% occurred (complete destruction of the organic skeleton [26,31,32,33]). Finally, at 476 °C and 838 °C, two small exothermic peaks were observed, associated with a small loss of mass close to 1.0% (both peaks were associated with the combustion of residual carbon and the decomposition of oxycarbonates of mixed metals, respectively, resulting in the formation of the stable phase of the desired compound [26,31,32]).

**Figure 4:**
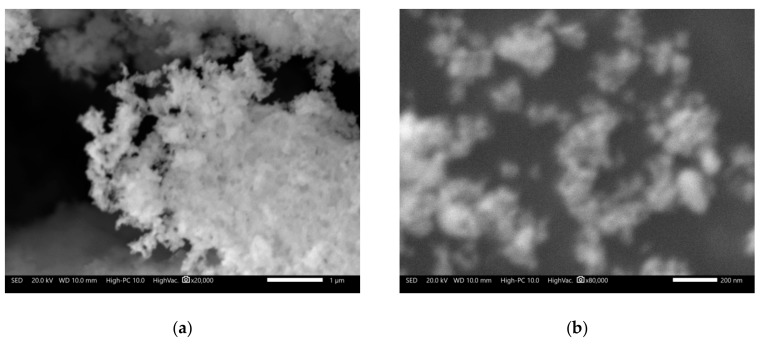
Morphological appearance of the catalyst calcined at 800 °C for 24 h. (**a**) 20,000X, (**b**) 80,000X.

Based on the previous analysis and preliminary calcination tests, the catalyst used in the transesterification process was obtained via pre-calcining the precursor gel at 200 °C for 2 h, and then calcined at 800 °C for 24 h in static air. Figure 4 shows the morphological aspects of the catalyst, and the presence of structures formed by particles smaller than 200 nm are observed. According to the XRD analysis, the identified compound corresponded to NdAlO_3_ with a completely crystalline structure. The specific surface area of the catalyst was 32 m^2^/g, and had an average pore size of 13.1 nm. The basic strength and basicity of the catalyst were *H*_ = 7.2–9.8 and 1.13 mmol/g, respectively. Although as individual oxides (Nd_2_O_3_ and Al_2_O_3_), their basic strengths were low (< 7.2), which can be increased when their surfaces are modified by the impregnation method with stronger bases [4,28], the measurements indicate that in the form of mixed oxides, their basicity properties improved.

### 3.2. Canola Oil Characterization

**Figure 5:**
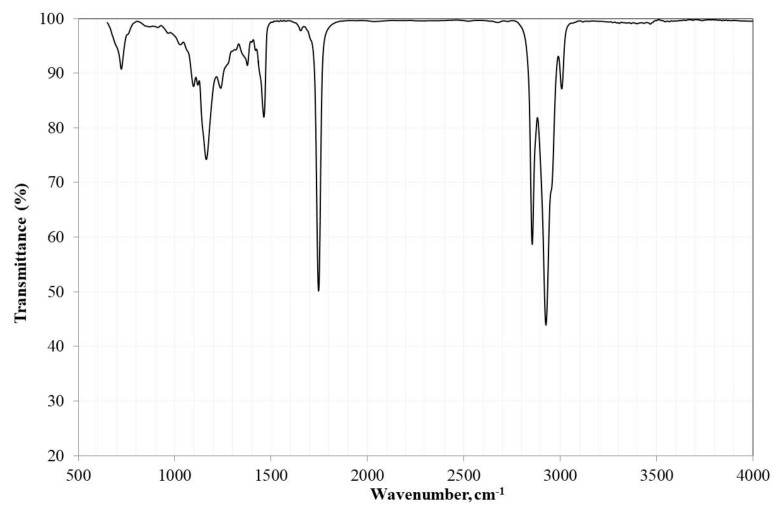
FTIR spectrum of edible canola oil.

Figure 5 shows the FTIR spectrum of the edible grade canola oil. The spectrum was similar to many FTIR spectra of vegetable oils. This type of analysis allowed for identifying the functional groups and the bands corresponding to various vibrations of stretching and bending of the oil. The FTIR spectrum analysis showed the main characteristic signals, such as: a peak at 3007 cm^−1^ attributed to the stretching vibration of *cis*-olefin double bonds (=C–H), an asymmetric stretch band of methylene at 2925 cm^−1^, a symmetric stretch band of methylene at 2854 cm^−1^, the band corresponding to the group C=O of the triglycerides at 1746 cm^−1^, the band corresponding to the group CH_2_ at 1462 cm^−1^, and the stretch corresponding to –COO at 1163 cm^−1^. This was in addition to other signals, such as the one at 723 cm^−1^ due to the overlap of the methylene equilibrium vibration, and the flexural vibration outside the plane of the *cis*-disubstituted olefins (HC=CH–) [34,35,36,37].

**Figure 6:**
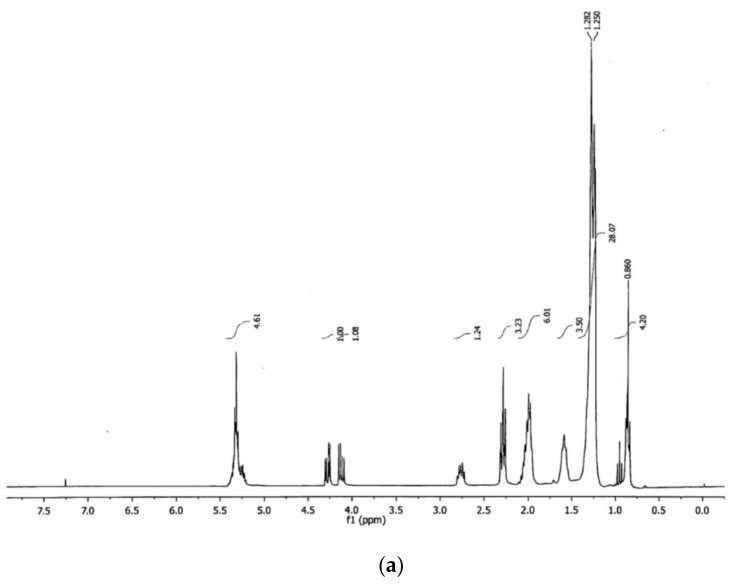

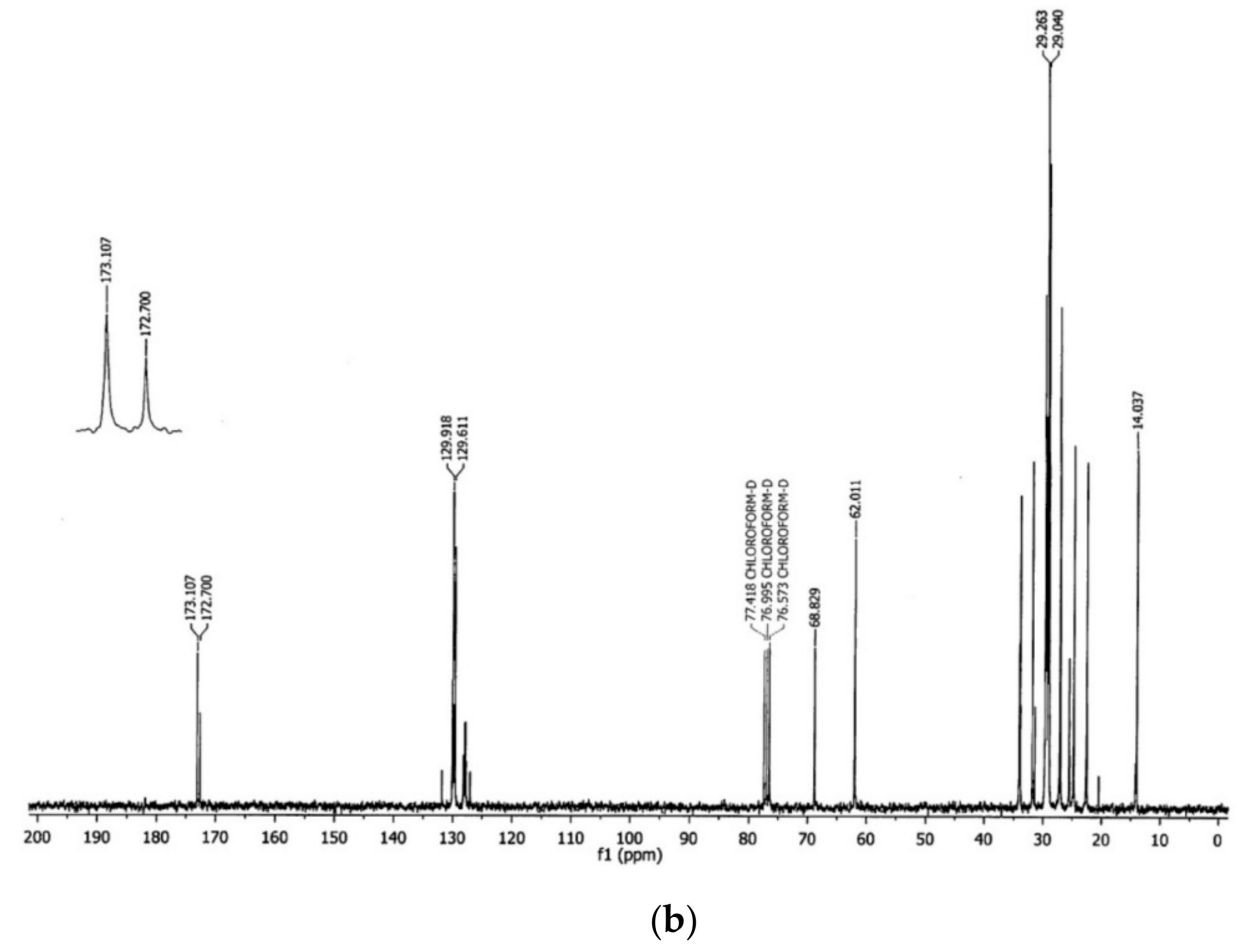
NMR spectra of canola oil: (**a**) ^1^H and (**b**) ^13^C (solvent used: CDCl_3_).

Figure 6 shows the ^1^H and ^13^C NMR spectra of the canola oil. The ^1^H NMR spectrum showed the main characteristic signals that have been reported for vegetable oils, with the intensity of them varying according to the type of acyl groups present. The general assignment of the ^1^H spectral signals is well documented and those found in this study completely corresponded to those reported in the literature [38]. From the 10 main characteristic bands, 8 corresponded to the acyl chains and 2 to the glyceryl group. The signals of the acyl chains were located at: 0.83–0.93 ppm, 0.93–1.03 ppm, 1.22–1.42 ppm, 1.52–1.70 ppm, 1.94–2.14 ppm, 2.23–2.36 ppm, 2.70–2.84 ppm, and 5.26–5.40 ppm, and corresponded to the functional groups: –CH_3_ of the saturated, oleic, and linoleic chains; –CH_3_ of the linolenic chains; –(CH_2_)_n_; –OCO–CH_2_–CH_2_-; –CH_2_–CH=CH–; –OCO–CH_2_-; =HC–CH_2_–CH=; and –CH=CH–, respectively. Glyceryl group signals were located at 4.10–4.32 ppm and 5.20–5.26 ppm, and they corresponded to the functional groups –CH_2_OCOR and –CHOCOR, respectively.

On the other hand, the ^13^C NMR spectrum showed the main resonances reported for vegetable oils, where the intensity varied according to the different acyl groups present. The general assignment of the signals was in accordance with those reported in the literature [39], namely, the main signals of interest were located at: 14.6–13.4 ppm, 20.9–20.3 ppm, 23.4–21.9 ppm, 25.3–24.3 ppm, 27.9–26.6 ppm, 30.7–28.2 ppm, 31.7–31.2 ppm, 32.3–31.7 ppm, 33.1–32.3 ppm, 34.9–33.3 ppm, 62.7–61.4 ppm, and 69.5–68.5 ppm, attributed to functional groups: CH_3_, ω-2 C in *n*-3 polyunsaturated acyl chains, others ω-2 C, C3 in acyl chains, allylic C (*cis*), –CH_2_–, ω-3 C in *n*-6 polyunsaturated acyl chains, other ω-3 CH_2_, allylic C (*trans*), C2 in acyl chains, α-carbon of the glycerol backbone, and β-carbon of the glycerol backbone, respectively.

It has been indicated that the carbons of the triacylglycerol chains show chemical shifts according to their positional distribution (*sn*-1,3 or *sn*-2) and degree of unsaturation, and these chemical shifts occur between 172 and 174 ppm [40]. According to the above, it was observed that the majority acyl group was oleic (C18:1), completely occupying positions *sn*-1 and *sn*-3, and mostly the *sn*-2 position of the glycerol backbone, in addition to the linoleic (C18:2) and linoleic (C18:3) in smaller proportion [41].

GC-MS analyses confirmed that the main fatty acids corresponded to the aforementioned. Table 1 shows the chemical composition of the canola oil used for the synthesis of biodiesel. Values were consistent with data reported in the literature [42]; however, its content of unsaturated fatty acids was slightly higher than the average reported.

### 3.3. Catalytic Reaction of Transesterification

Unlike the typical homogenous transesterification process, in heterogeneous transesterification, the reactants form a three-phase system (oil–methanol–catalyst) where the diffusion resistance is the limiting step in the synthesis of biodiesel [4]. In order to reduce the limitations of mass transfer, some studies have proposed the use of co-solvents to improve the oil–methanol miscibility [5].

Although it has been reported that in any catalytic process, the reduction in catalyst particle size, as well as an increase in its surface area, causes an increase in the reaction rate and an improved performance [1], previous transesterification tests of this study indicated that under relatively mild reaction conditions (65 °C, 1 atm), the catalyst was practically inactive regardless of the oil–methanol–catalyst ratio. This was possibly due to two factors, namely, the poor dispersion of the catalyst into the oil–methanol system, and the zero solubility of the oil in the methanol. In addition, because the transesterification is an endothermic reaction, a higher temperature is necessary in order for the reaction to be carried out appreciably [12].

Therefore, it was decided to carry out the transesterification tests by increasing the temperature, and consequently the pressure, of the system. This was because it has been reported that by increasing both the reaction temperature, and as a consequence, the reactor pressure, the biodiesel yield increases significantly [1]. In addition, the increase in the transesterification temperature improves the oil–methanol miscibility and increases the reaction rate [15] because the mass transfer resistance of the transesterification process is reduced. Taking into account the test temperatures (100, 150, and 200 °C), and according to the Antoine expression, the methanol vapor pressure is approximately 3.5, 13.7, and 38.9 bar, respectively. This combination of high temperature and high pressure will favor the transesterification reaction [15].

**Figure 7:**
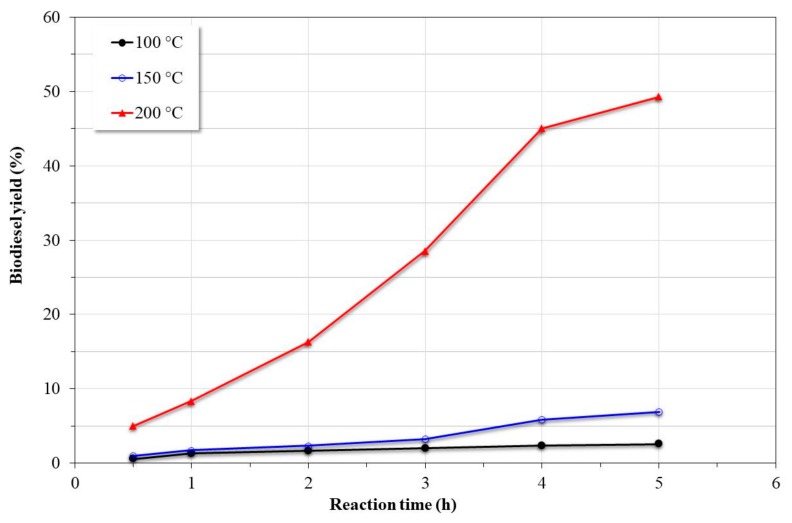
Effect of temperature on the catalytic reaction of transesterification (methanol:oil mass ratio of 2:1 and 10% catalyst with respect to oil, both as weight ratios).

Figure 7 shows the temperature effect on the catalytic reaction of the transesterification of canola oil with a methanol:oil mass ratio of 2:1 and 10% catalyst with respect to oil, both as weight ratios. From the graph, it was observed that the transesterification reaction increased by increasing the reaction temperature; however, at temperatures of 100 and 150 °C, the yield of biodiesel was less than 10%. The remarkable increase in the yield of biodiesel obtained at 200 °C (≈50%) may have been due to the high vapor pressure generated (≈38.9 bar), which improved the oil–methanol solubility by reducing the mass transfer resistance [15]. The increase in the miscibility of the reactants favored the formation of the active nucleophile (CH_3_O–) as a consequence of the methanol deprotonation onto the basic sites of the catalyst, which has been established as the general mechanism of transesterification in the presence of a solid basic catalyst [1].

Theoretically, 3 mol of methanol are required to achieve the transesterification of 1 mol of triacylglyceride. However, because this process is reversible, an excess of methanol is required for the reaction to be carried out favorably. Notwithstanding the above, it is indicated that too much methanol decreases the conversion [12].

**Figure 8:**
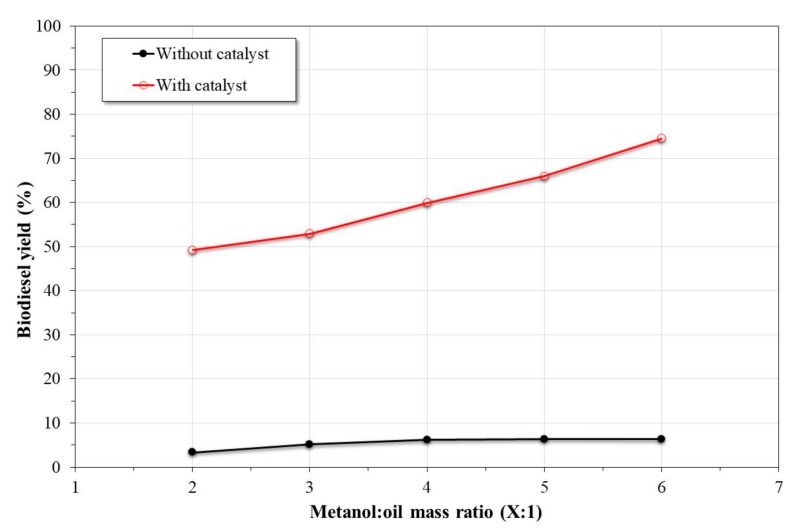
Effect of the methanol:oil ratio on the biodiesel yield in the presence and absence of catalyst (reaction time of 5 h at 200 °C).

Figure 8 shows the effect of the methanol:oil ratio on the biodiesel yield obtained both in the presence and absence of a catalyst. According to the graph, it was observed that in the absence of a catalyst, the biodiesel yield was very low, regardless of the methanol:oil ratio used. Similar observations have been reported in the transesterification of other oils, where non-catalytic transesterification can be neglected at temperatures of 200 °C and below [5,19,43]. However, in the presence of a catalyst, it was observed that the biodiesel yield improved by increasing the methanol:oil ratio, where at the maximum evaluated ratio, the biodiesel yield was around 75%. Although it has been reported that an excess in methanol concentration can affect the biodiesel yield due to a decrease effect in the oil and catalyst concentration, in this study, the opposite effect was observed [1]. This can be due to an increase in the miscibility of the oil and methanol as a consequence of the high reaction pressure, which reduced the mass transfer resistance.

**Figure 9:**
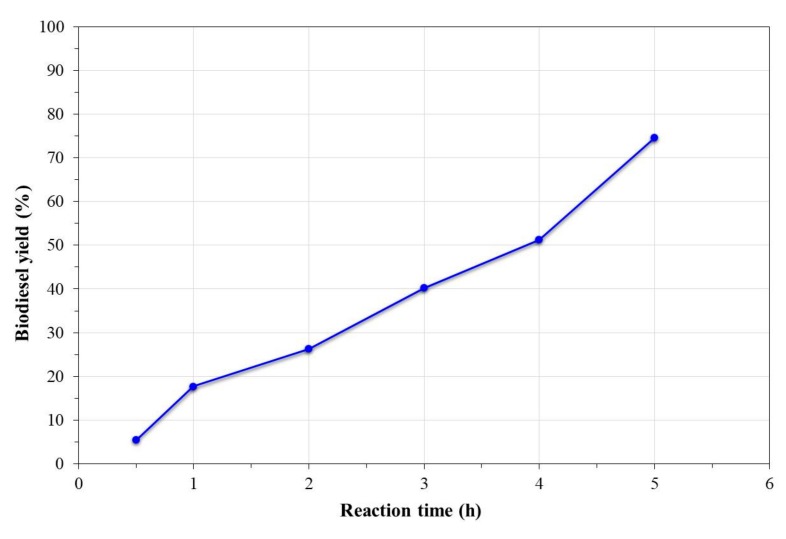
Effect of the reaction time on the transesterification reaction of canola oil.

Figure 9 shows the effect of the reaction time on the biodiesel yield obtained at the optimum conditions of transesterification, methanol:oil mass ratio of 6:1, and temperature of 200 °C. From the graph, it was observed that in the reaction time evaluated, the conversion kinetics were linear; this indicated that at higher reaction times, the biodiesel yield could increase or reach the steady state at higher yields than the last value obtained. As already described, this tendency could be due to the high reaction pressure that favored the oil:methanol miscibility and the contact with the catalyst, thus reducing the mass transfer resistance.

### 3.4. Recyclability of the Catalyst

**Figure 10:**
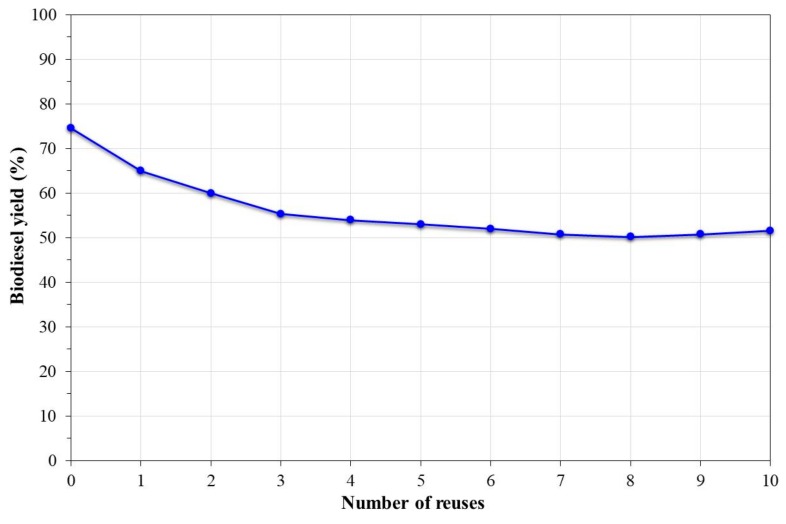
Effect of the reuse of the catalyst on the biodiesel yield.

Two factors are important in order to define the viability of a catalyst for the synthesis of biodiesel: its recyclability and stability [44]. Figure 10 shows the biodiesel yield obtained based on the number of reuses of the catalyst at a methanol:oil mass ratio of 6:1 and a reaction time of 5 h at 200 °C. From the graph, it was observed that there was a constant decrease in the activity of the catalyst until six reuses, and then this remained constant at around 50% conversion. This implied a reduction of 34% of the catalytic activity of the catalyst. The decrease in catalytic activity has been associated with the loss of active sites on the catalyst’s surface [1,19].

**Figure 11:**
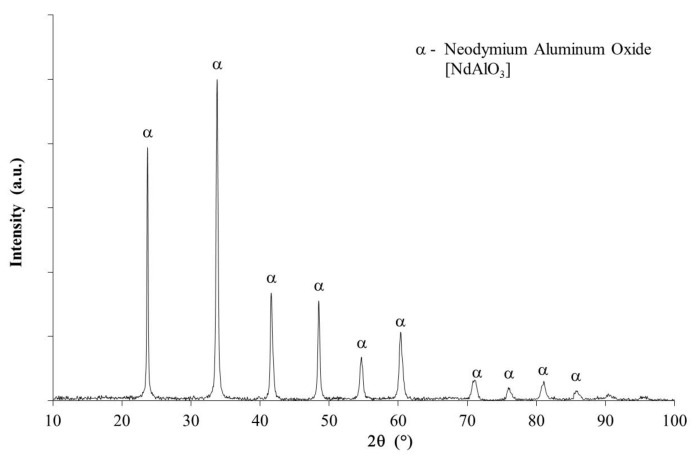
X-ray diffraction spectrum of the catalyst after 10 cycles of transesterification and reactivation via calcination at 600 °C in each cycle.

Figure 11 shows the XRD spectrum of the catalyst after 10 cycles of transesterification. According to the XRD spectrum, it was observed that the catalyst was free of contamination and retained a completely crystalline structure corresponding to the NdAlO_3_ perovskite, identical to that obtained when it was synthesized.

**Figure 12:**
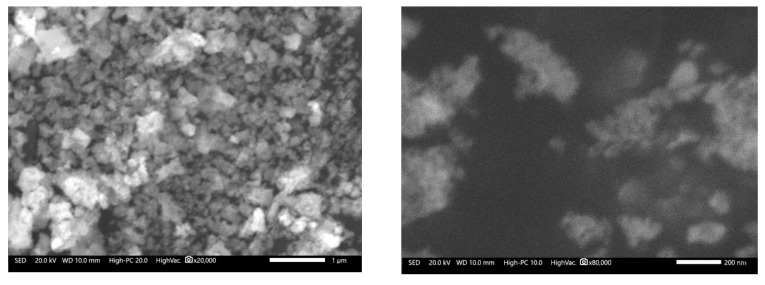
Morphological aspect of the catalyst after 10 reuses.

Figure 12 shows the morphological aspects of the catalyst after 10 cycles of reuse. According to the micrographs, it was observed that the catalyst presented morphologies similar to those observed in the newly synthesized catalyst (Figure 4), with the only visible difference being the formation of larger nanoparticle clusters. The specific surface area of the reused catalyst was 24 m^2^/g and the average pore size was 10.1 nm. It was clear that these values were lower than those determined for the catalyst before being used. These reductions were expected due to the high pressures reached in the reactor, thereby decreasing the catalytic activity of the nanoparticles. Other factors that affect the catalytic activity of catalysts involving rare earth oxides are the presence of moisture and CO_2_ due to the formation of compounds such as Ln(OH)_3_, LnOH(CO_3_), and Ln_2_O_2_(CO_3_) [5,12,19], and in general, the glycerin formed has a strong effect on the catalytic activity because it can block the catalytic sites [18].

### 3.5. Analysis of Reaction Products

The following results were obtained after analyses were performed on the reaction products of the transesterification process of the canola oil after its physical separation from the reaction mixture as indicated in the experimental procedure section. The reaction products were not subjected to any purification process.

**Figure 13:**
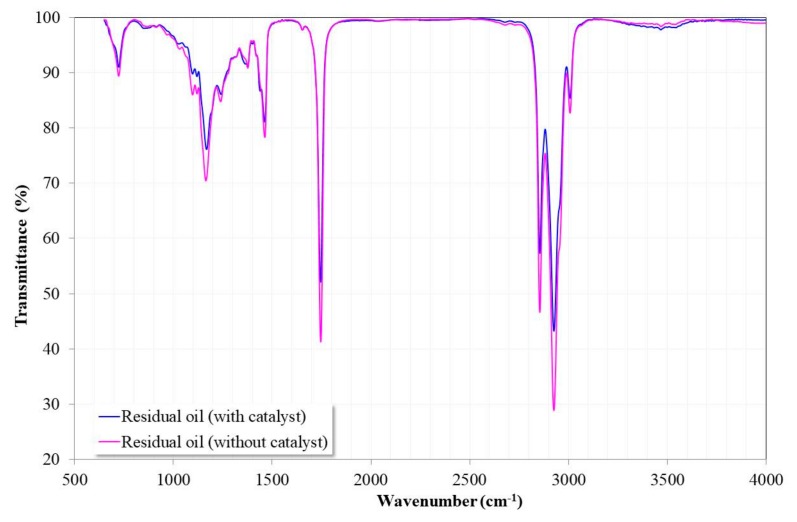
FTIR spectra of the residual canola oil after the transesterification process with and without the catalyst.

Figure 13 shows the FTIR spectra of the residual canola oil after the transesterification process in the presence and absence of the catalyst at 200 °C for 6 h. It can be seen that both spectra were identical to the canola oil used for the synthesis of biodiesel (Figure 5). This indicated that the oil did not experience additional degradation reactions due to the temperature conditions of the transesterification reaction.

However, in the case of residual oil in the presence of the catalyst, it was possible to observe a broad shoulder at 3467 cm^−1^. In the case of residual oil without the catalyst, this band was smaller. It has been indicated that its presence may correspond to the alcohol group (OH), oxydryl (OH–), or hydrogen bonding [11,45]. The presence of this signal can be associated with the alcohol groups of the glycerol, that is, mono- and diacylglycerides are present in the oil due to the incomplete transesterification of the triacylglycerides. Due to the higher reaction rate, it is understandable that in this case, their presence was detected.

**Figure 14:**
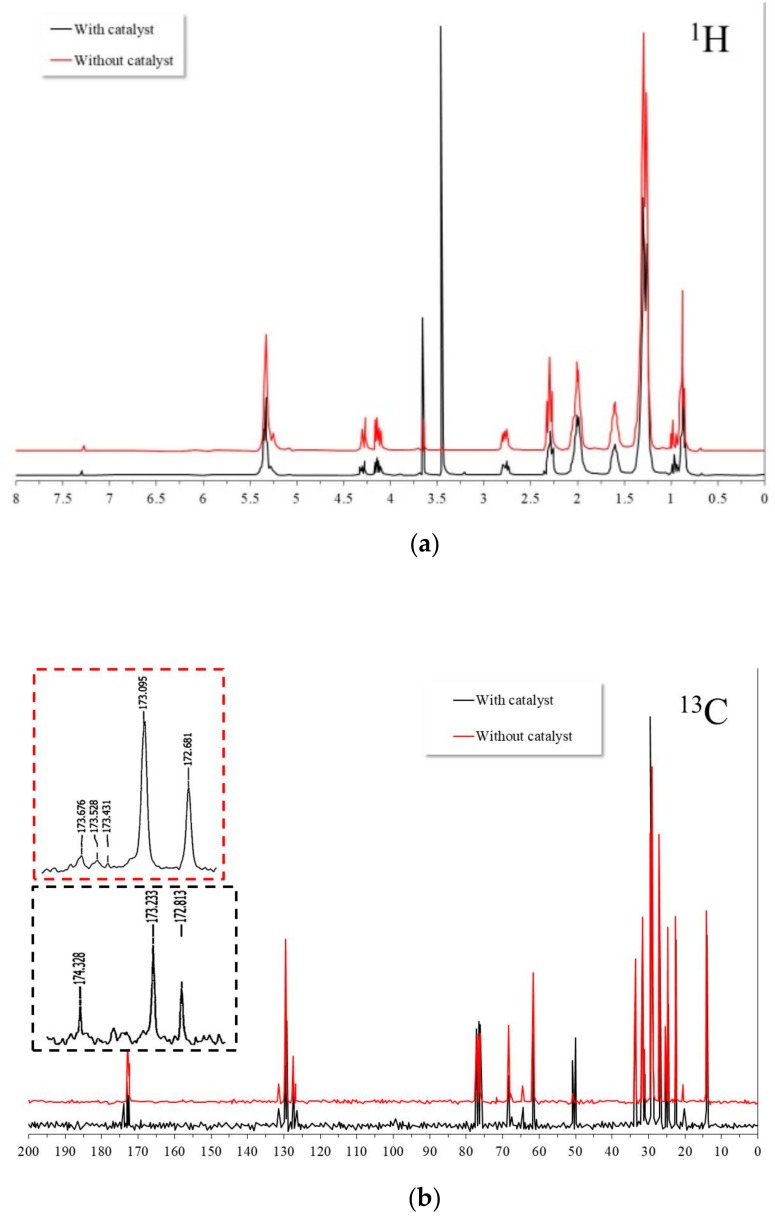
NMR spectra of the residual canola oil after the transesterification process with and without the catalyst: (**a**) ^1^H and (**b**) ^13^C (solvent used: CDCl_3_).

Figure 14 shows the NMR spectra of protons from ^1^H and ^13^C of the residual oil after the transesterification process with and without the catalyst present at 200°C for 6 hours. It can be observed that in both cases, the spectra showed the same signals observed in the source oil discussed previously. However, from the ^13^C NMR spectra, it was possible to observe differences in the signals of the acyl group region (172–174 ppm). In the case of residual oil in the catalyst absence, the new observed signals suggested that during the transesterification process, mono- and di-acylglycerides were formed as reaction byproducts. In addition, a rearrangement of the acyl groups in the glycerol backbone possibly occurred due to steric hindrance effects. Similar effects have been observed in both the enzymatic and chemical hydrolysis of vegetable oils [40]. On the other hand, in the case of residual oil in the catalyst’s presence, the observed signals were different. In this case, the signals were located at higher frequencies. It was possible to infer the formation of mono-, di-, and triglycerides with different conformations of the acylgroups at positions *sn*-1,2,3. Apparently, the new acyl groups were formed by the minor fatty acids present in the source canola oil.

**Figure 15:**
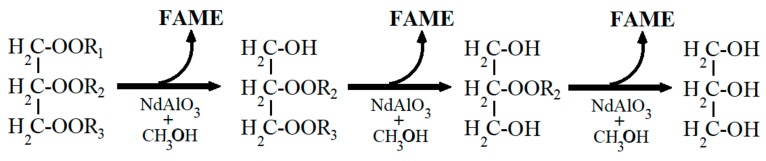
Schematic transesterification of canola oil with methanol and NdAlO_3_ as the catalyst (FAME = fatty acid methyl esters).

The above was confirmed using the GC-MS analyses. Table 2 shows the fatty acid composition of the residual oil in the presence and absence of the catalyst. Due to the low level of conversion in the absence of the catalyst, the fatty acid composition of the residual oil was very similar to that of the source oil. A lower content of the main fatty acid (oleic acid) was observed, and therefore, an increase in the concentration of the remaining fatty acids. However, the fatty acid composition of the residual oil in the presence of the catalyst was completely different, which was congruent with its ^13^C NMR spectrum. A reduction in the concentration of the majority fatty acid of approximately 90% was observed, along with an increase in the concentration of the main minority fatty acids. It has been indicated according to the ^13^C NMR spectrum of canola oil, that the acyl oleic group occupied positions *sn*-1 and *sn*-3 of the glycerol backbone. As such, this may suggest that the transesterification reaction started in those positions, and that the residual oil was mainly composed of mono and di-acylglycerides, as was suggested by its FTIR spectrum. In addition, because the transesterification reaction is reversible, it was possible that a reorganization of the hydrocarbon chains also occurred. Based on the foregoing, it can be concluded that the transesterification reaction in the presence of NdAlO_3_ occurred according to the illustration in Figure 15.

**Figure 16:**
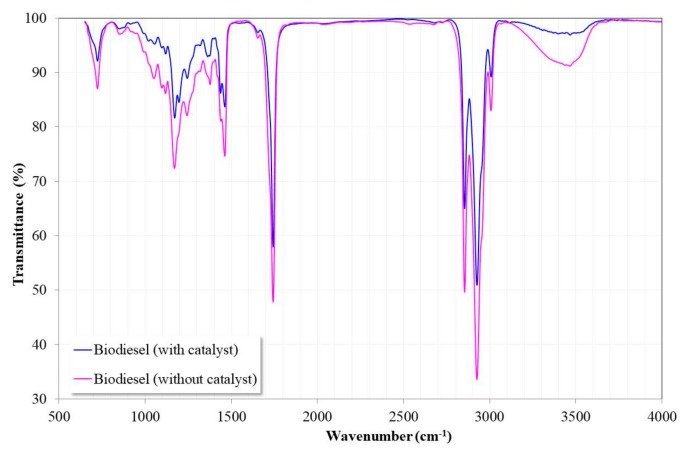
FTIR spectra of the biodiesel synthesized with and without the catalyst.

Figure 16 shows the FTIR spectra of the biodiesel synthesized in the presence and absence of the catalyst at 200 °C for 6 h. Due to the structural similarity that exists between triglycerides and methyl esters, the spectra of both are similar [11]. In the spectra, the characteristic peak of the carbonyl group of the methoxyesters (–CO–OCH_3_) was observed at ≈1743 cm^−1^, which has been used to confirm the conversion of the oil to biodiesel [45,46]. The only visible difference in both spectra was the presence of a broad band associated with the glycerol alcohol group at ≈3465 cm^−1^. This suggested the presence of mono- and diacylglycerides formed during the transesterification process, and their presence was lower in the biodiesel synthesized with the catalyst.

**Figure 17:**
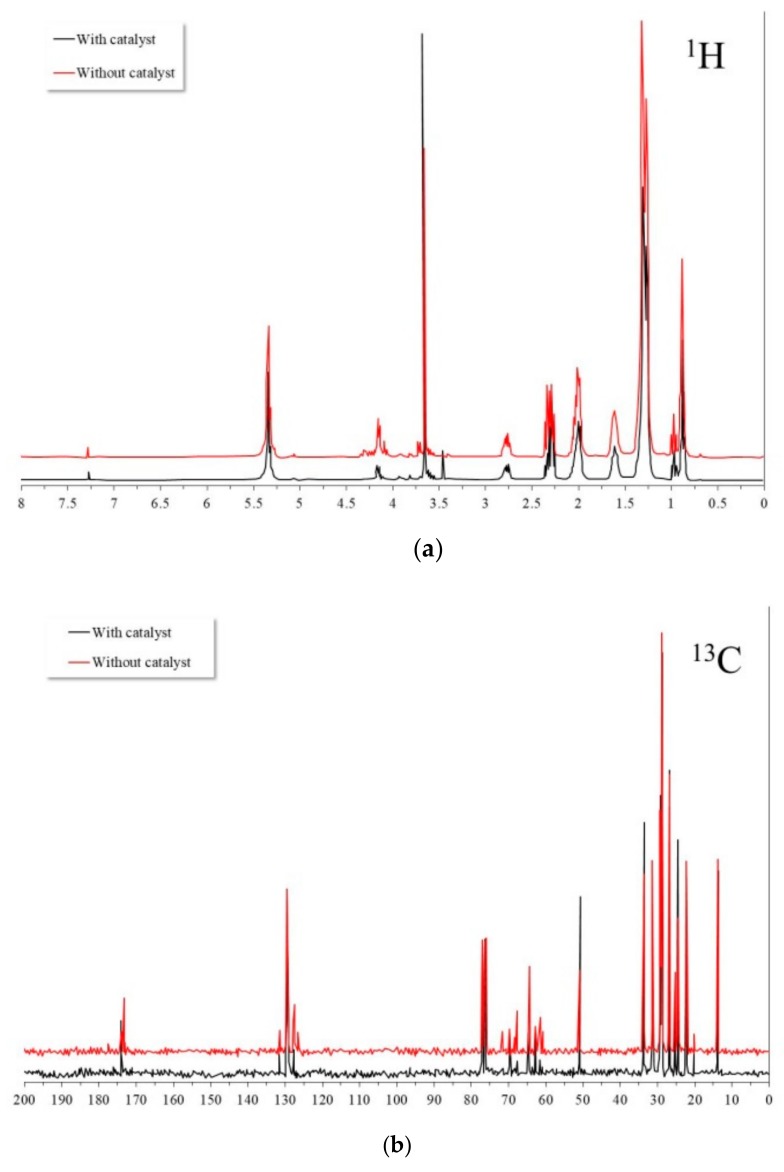
NMR spectra of the biodiesel synthesized with and without the catalyst: (**a**) ^1^H and (**b**) ^13^C (solvent used: CDCl_3_).

Figure 17 shows the NMR spectra of protons from ^1^H and ^13^C of the biodiesel synthesized with and without the catalyst present at 200 °C for 6 h. In both cases, the spectra are similar. In the case of the ^1^H NMR spectra, at frequencies lower than 3 ppm, a great coincidence was observed with the signals observed in the canola oil (Figure 6a). The main difference was the characteristic peak of methoxyl protons (–OCH_3_) of the methyl ester moiety observed as a singlet at 3.661 ppm, which together with the triplet of protons α-CH_2_ at 2.26 ppm, are the two peaks that confirm the existence of methyl esters [45,46,47]. In the case of biodiesel synthesized with a catalyst, the dominant presence of the singlet of the methoxyl protons characteristic of the methyl ester moiety was observed. Other peaks observed were a triplet at 0.88 ppm related to methyl terminal protons (–CH_3_); a strong signal at 1.27 ppm related to methylene protons of the carbon chain; a multiplet at 1.58 ppm related to the protons of β-carbonyl-methylenes; a triplet at 2.3 ppm related to α-carbonyl-methylenes; and at 2.0, 2.8, and 5.28 ppm, signals associated with unsaturations related to allylic, bis-allylic, and olefinic hydrogens, respectively [46,47]. The absence of signals beyond 5.5 ppm indicated the absence of aliphatic and oleic hydrogens [48].

Similarly, the ^13^C NMR spectra showed much similarity to that of the canola oil (Figure 6b). It has been reported that carbon signals were grouped in five main spectral regions [45,49]. These regions involved the carbonyl atom region (–COOR) detected at ≈175 ppm, the region of carbon atoms associated with double bonds (–C=C–) at ≈130 ppm, the carbon atoms of the methyl ester groups (–OMe) at ≈50 ppm, the methylene group region at ≈20–35 ppm, and the terminal methyl group region (–CH_3_) of the hydrocarbon chains at ≈14 ppm. Furthermore, the presence of the characteristic peaks corresponding to the carbonyl ester (–COO–) and C–O were found at 172–175 and 51.35 ppm, respectively, which confirmed the presence of the methyl esters [46,48]. Additionally, the peaks between 127–132 ppm indicated the insaturations in the methyl esters, the peak in 14 ppm was due to the terminal carbon of the methyl groups, and the signals between 22–34 ppm were related to the long chain methylene carbons of the methyl esters of fatty acids [46,48].

Table 3 shows the chemical composition of biodiesel synthesized in the presence and absence of the catalyst. In general, it could be observed that the fatty acid content of the hydrocarbon chains of the methyl esters was similar to each other and in accordance with the analysis of the source oil (Table 1).

**Figure 18:**
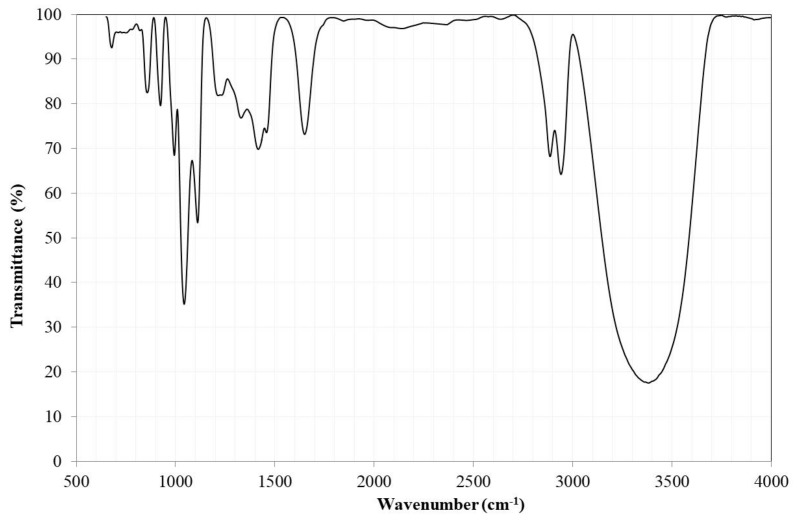
FTIR spectrum of crude glycerol obtained as a reaction byproduct.

Figure 18 shows the FTIR spectrum of the crude glycerol obtained as a reaction byproduct of the transesterification process via heterogeneous catalysis of the canola oil at 200 °C for 6 h. The FTIR spectrum was identical to others reported in the literature [14,50]. It was possible to identify the peaks assigned to the superposition of the C–H and OH bending planes of the glycerol molecule (≈1200–1500 cm^−1^), in addition to the presence of the OH group at ≈3500–3000 cm^−1^ and ≈1650 cm^−1^ [50]. This showed the high purity of the crude glycerol obtained by the heterogeneous transesterification process. This is a great advantage if it is considered that in the case of the homogeneous transesterification process, the crude glycerol may contain methanol, water, salts, free fatty acids, soaps, methyl esters of fatty acids, and glycerides. This has meant that due to the high cost of its purification process, it is preferable not to recover it [13,14]. Therefore, the synthesis process of biodiesel via heterogeneous transesterification offers a great advantage by reducing the production costs due to the high purity of the products and byproducts generated.

Previous studies have shown that both Nd_2_O_3_ [4] and Al_2_O_3_ [19,28,43] as individual oxides have negligible catalytic activity under normal transesterification conditions (60–65 °C). However, its superficial modification (impregnation method) increases its catalytic activity [4,19,28,43]. Similarly, an increase in the transesterification temperature also increases its catalytic activity.

Studies by Russbueldt et al. [19] have demonstrated conversion efficiencies close to 70% in the case of Nd_2_O_3_, and 15% in the case of Al_2_O_3_, when the temperature increases to 200 °C. Similarly, when Nd_2_O_3_ is supported on Al_2_O_3_, its conversion efficiency is similar to that of pure Nd_2_O_3_. However, the catalytic activity of both in the form of mixed oxides (NdAlO_3_) is reported to be much lower than that of pure Nd_2_O_3_ in the experimental conditions studied by the authors (1:1:0.1 mass ratio of oil:methanol:catalyst) [19]. The reasons for the low catalytic activity of NdAlO3 were attributed to the high crystallinity of the compound and its low surface area due to the synthesis method used (flash combustion synthesis). As such, according to the results of this study, it can be seen that the synthesis method used (sol-gel) caused the greater catalytic activity of NdAlO_3_ due to the greater surface area of the nanoparticles. Additional studies carried out with Nd_2_O_3_ and Al_2_O_3_ (both with an average particle size of 1 micron) as catalysts under the same experimental conditions in which NdAlO_3_ was evaluated yielded similar efficiency values as those from Russbueldt et al. [19]. In this case the Nd_2_O_3_ showed an efficiency of around 68%, and around 9% for the Al_2_O_3_.

Therefore, based on the results of this study, it was shown that NdAlO_3_ nanoparticles can be used as a catalyst for the synthesis of biodiesel with slightly higher yields than Nd_2_O_3_ simply by reducing the particle size and increasing the concentration of methanol.

## 4. Conclusions

In this work, the catalytic capacity of NdAlO_3_ nanoparticles in the synthesis of biodiesel was evaluated. The nanoparticles were synthesized using the sol-gel process, and its thermal treatment created a pure NdAlO_3_ compound with a particle size in the order of 100 nm. It was found that increasing the reaction temperature increased the biodiesel yield, and this could be attributed to the increase in the reactants’ miscibility. The increase in the miscibility decreased the mass transfer resistance, and the contact with the active sites of the catalyst was favored. Similarly, increasing the concentration of methanol increased the yield of biodiesel; this effect was possibly due to an increase in the concentration of the solubilized methanol. Similarly, by increasing the reaction time, a linear increase in the biodiesel yield as a function of time was observed. The catalyst showed a reduction of 34% in its catalytic activity up until six reuses; beyond this, its activity remained constant with a 50% biodiesel yield. Although the catalyst showed no changes in its crystalline structure, the activity loss could be due to the formation of nanoparticle clusters, possibly due to the high reaction pressures reached. It was observed that the residual oil found after the catalytic transesterification process showed a chemical composition completely different to that of the source canola oil. The change in the chemical composition of the residual oil could be due to the reversibility of the transesterification reaction, or because the reaction was favored at positions sn-1 and sn-3 of the glycerol backbone. In addition, both crude biodiesel and crude glycerol were produced with a high purity.

## Figures and Tables

**Table 1:** Fatty acid composition of canola oil (% by weight).

Palmitic (16:0)	Estearic (C18:0)	Oleic (C18:1)	Linoleic (C18:2)	Linolenic (C18:3)	Arachidic (C20:0)	Eicosenoic (C21:1)	Behenic (C22:0)
1.62	0.68	66.53	20.95	8.57	0.27	1.13	0.24

**Table 2:** Fatty acid composition of the residual oil (% by weight).

	Palmitic (16:0)	Estearic (C18:0)	Oleic (C18:1)	Linoleic (C18:2)	Linolenic (C18:3)	Arachidic (C20:0)	Eicosenoic (C21:1)	Behenic (C22:0)
Residual oil without catalyst	4.16	1.68	60.85	21.93	8.20	1.05	1.68	0.44
Residual oil with catalyst	57.30	34.29	8.40	—	—	—	—	—

**Table 3:** Fatty acid composition of the residual oil (% by weight).

	Palmitic (16:0)	Estearic (C18:0)	Oleic (C18:1)	Linoleic (C18:2)	Linolenic (C18:3)	Arachidic (C20:0)	Eicosenoic (C21:1)	Behenic (C22:0)
Biodiesel without catalyst	4.55	2.12	63.60	20.44	6.83	2.46	—	—
Biodiesel with catalyst	3.65	1.56	63.70	20.33	8.57	0.94	1.06	0.19
